# Validation of the Italian consensus recommendations for the biomarker‐based etiological diagnosis of mild cognitive impairment: study design and preliminary data

**DOI:** 10.1002/alz.088884

**Published:** 2025-01-09

**Authors:** Claudio Singh Solorzano, Cristina Festari, Stefania Orini, Eleonora Castagna, Matteo Cotta Ramusino, Fabrizia D'Antonio, Adolfo Di Crosta, Alessia Masòtino, FEDERICO MASSA, Alice Mazzonetto, Massimiliano Panigutti, Noemi Ravì, Michela Pievani, Laura Bonanni, Giuseppe Bruno, Annachiara Cagnin, Roberto Gatta, Giovanni B. Frisoni

**Affiliations:** ^1^ IRCCS Istituto Centro San Giovanni Di Dio Fatebenefratelli, Brescia Italy; ^2^ Università degli Studi di Brescia, Brescia Italy; ^3^ Sapienza University of Rome, Roma Italy; ^4^ IRCCS Mondino Foundation, Pavia Italy; ^5^ Sapienza University of Rome, Rome, Rome Italy; ^6^ University G. d'Annunzio of Chieti‐Pescara, Chieti Italy; ^7^ University of Genova, Genova Italy; ^8^ IRCCS Ospedale Policlinico San Martino, Genova Italy; ^9^ University of Padova, Padova Italy; ^10^ Geneva University Hospitals, Geneva Switzerland; ^11^ University of Geneva, Geneva Switzerland

## Abstract

**Background:**

In 2020, five Italian scientific societies issued diagnostic recommendations to improve the rational and combined use of biomarkers in the clinical practice of Italian memory clinics (Boccardi et al., 2020). The present project will aim to compare the most frequent diagnostic workups in three academic memory clinics before (pre‐recommendations: January 2018 ‐ December 2019) and after (post‐recommendations: October 2022 ‐ October 2023) the release of the recommendations to assess their implementation in clinical routine. Preliminary results on pre‐recommendations data are presented here.

**Method:**

We retrospectively reviewed the medical charts of consecutive patients referred to the academic memory clinics of Padova, Chieti, and Roma for a first visit in the pre‐recommendations period. The collected information included socio‐demographic data, number and dates of clinical visits and biomarker exams (i.e., FDG‐, amyloid‐, tau‐PET, cerebrospinal fluid [CSF], DAT SPECT/PET, and cardiac MIBG scintigraphy). A diagnostic hypothesis was extracted for each clinical visit until the final diagnosis.

**Result:**

Since October 2022, 500 patients’ medical charts were reviewed. Patients were divided into three groups: 148 (30%) patients came to clinical observation not for diagnostic purposes (e.g., second opinion, disability claim), 242 (48%) received a diagnosis based on neuropsychological assessment and morphological imaging only, and 110 (22%) were in a biomarker‐based diagnostic pathway. The latter group included 31 patients with Alzheimer’s disease (AD), 19 with frontotemporal lobar degeneration (FTLD), 28 with a Lewy body spectrum disease (LBD), and 32 with non‐neurodegenerative aetiology or vascular dementia. Biomarker‐based diagnoses of AD, FTLD and non‐neurodegenerative diseases were commonly based on FDG‐PET (AD: 77%, FTLD: 84%, non‐neurodegenerative: 66%) and CSF (52%, 37%, and 44%). In LBD (n = 28), the first‐choice biomarker was FDG‐PET (61%), followed by DAT SPECT/PET (39%).

**Conclusion:**

This preliminary analysis outlines the most frequent diagnostic pathways, providing the prevalence of the use of biomarkers in Italian academic memory clinics in the pre‐recommendations period. Once the post‐recommendations data collection is completed, we will evaluate whether the recommendations are feasible and usable in real‐world clinical workups by applying process‐mining techniques (Gatta et al., 2020) to estimate the prevalence and type of deviations from the ideal pathway based on recommendations.

